# Intestinal Amoebiasis Associated With Inflammatory Bowel Diseases: An Eight-Year Retrospective Study at Ibn Sina University Hospital, Rabat, Morocco (2014-2022)

**DOI:** 10.7759/cureus.86866

**Published:** 2025-06-27

**Authors:** Fatima Zahra Lfaquir, Imane Zouaoui, Khalil Zimi, Sarra Aoufi

**Affiliations:** 1 Central Laboratory of Parasitology and Mycology, Ibn Sina University Hospital, Faculty of Medicine and Pharmacy, Mohammed V University, Rabat, MAR

**Keywords:** chronic inflammatory bowel disease, crohn`s disease, entamoeba histolytica infection, intestinal amoebiasis, morocco, ulcerative colitis disease

## Abstract

Introduction

Amoebiasis, caused by the protozoan parasite *Entamoeba histolytica*, remains a significant public health concern in our country due to its endemicity. It poses a unique challenge in patients with inflammatory bowel disease (IBD), where its symptoms often mimic disease flare-ups, leading to diagnostic uncertainty and potential delays in appropriate management. The overlap in clinical presentation between amoebiasis and IBD exacerbations can result in misdiagnosis or inappropriate treatment, further complicating patient outcomes. In this context, our study was designed to explore and describe the epidemiological profile of intestinal amoebiasis specifically in the IBD patient population. By identifying patterns of co-infection, risk factors, and clinical manifestations, we aim to enhance early recognition and guide targeted diagnostic and therapeutic strategies. This is particularly important for improving outcomes and optimizing care in regions where amoebiasis remains endemic.

Materials and methods

This retrospective study examined 684 stool samples from IBD patients sent to the central parasitology-mycology laboratory at Ibn Sina University Hospital in Rabat between June 2014 and July 2022. A positive diagnosis was established through parasitological examination of stool (PES) samples using a combination of fresh examination and two concentration techniques: Merthiolate-Iodine-Formol and Bailenger.

Results

Of the 684 stool samples collected from patients with IBD, 162 tested positive, representing a prevalence rate of 24%. Of these, 78 were from patients with ulcerative colitis (UC), 67 with Crohn's disease (CD), and 17 with indeterminate IBD. Twenty patients were in remission at the time of the study: ten with UC, seven with CD, and three with unspecified IBD. The majority of patients were adults (159 cases), with a female predominance (sex ratio M:F = 0.7). In our study, intestinal parasitism showed a predominance of *Entamoeba histolytica* (60%), followed by the cystic form of the parasite (23%). Both forms were present in 14% of cases.

Conclusion

According to the literature, amoebiasis can mimic or exacerbate the clinical manifestations of IBD, complicating the differential diagnosis and increasing the risk of inadequate treatment in the absence of systematic PES samples. These results corroborate the findings of our study, which highlight the need for early diagnosis and appropriate management of IBD to prevent complications.

## Introduction

Intestinal amoebiasis is a parasitic disease affecting people worldwide. It is caused by *Entamoeba histolytica*, a protozoan parasite that resides in the human colon. The disease is defined as harboring this parasite, with or without symptoms [1]. Transmission primarily occurs via the fecal-oral route, through ingestion of infectious cysts present in contaminated water or food [2]. *E. histolytica* has two life cycle forms: the cyst form, responsible for human-to-human transmission, and the invasive, hematophagous trophozoite form, which colonizes the colon and causes tissue damage [3]. The pathogenesis relies on the trophozoites' ability to adhere to the intestinal mucosa through specific lectins, induce direct cytolysis of epithelial cells, and provoke a local inflammatory response [4].

Amoebiasis is asymptomatic in 80-90% of cases [5]. In symptomatic cases, it presents with symptoms resembling dysentery, including abdominal pain and bloody, mucous-filled diarrhea. Complications include necrotizing colitis, intestinal perforation, and toxic megacolon. The most frequent extraintestinal manifestation is an amebic liver abscess [4]. This broad clinical spectrum makes differential diagnosis challenging, especially in endemic areas where amoebiasis can mimic inflammatory bowel disease (IBD).

IBD includes Crohn’s disease (CD) and ulcerative colitis (UC). It is a chronic inflammatory disorder of the digestive tract with a multifactorial etiology involving genetic, immunological, and environmental factors [6]. The incidence of IBD is increasing markedly in developing countries, particularly in North Africa, reflecting an epidemiological transition toward chronic, Western-type diseases [7].

Clinically, IBD manifests as abdominal pain, chronic diarrhea (sometimes bloody), weight loss, marked fatigue, and an increased risk of long-term complications, including colorectal cancer [8]. A multidisciplinary approach is necessary for diagnosis, incorporating clinical, biological, endoscopic, and histopathological data. In endemic regions, however, the clinical presentation may resemble infections such as intestinal amoebiasis, which poses a significant diagnostic challenge [7].

The interaction between intestinal amoebiasis and IBD is of considerable clinical importance. The co-occurrence of these two conditions can mask or distort the initial diagnosis, which can have serious consequences if immunosuppressive treatments are initiated in patients harboring *E. histolytica* [9]. This exposes patients to a risk of parasitic dissemination and severe complications. Therefore, maintaining high clinical suspicion of this association is essential, especially in areas with high amoebic endemicity [10].

This study aims to describe the epidemiological profile of intestinal amoebiasis in patients with IBD, analyze their clinical and parasitological characteristics, and highlight the diagnostic and therapeutic challenges related to their co-occurrence. The study seeks to improve clinical management and reduce the risk of diagnostic errors or delays.

## Materials and methods

Type, location, and period of the study

This retrospective study was conducted at the Central Laboratory of Parasitology and Mycology at Ibn Sina University Hospital in Rabat. It covers an eight-year period from June 2014 to July 2022 and involves the analysis of 684 stool samples.

Inclusion and exclusion criteria

The study included adult and paediatric patients with IBD, who were either hospitalized or attending outpatient clinics at Ibn Sina University Hospital in Rabat. Only stool samples from these patients were selected for analysis. Samples from other patients or those with incomplete data were excluded.

Sample size

A total of 684 stool samples from patients meeting the inclusion criteria were analyzed. As this was a retrospective study, no a priori sample size calculation was performed. The sample size corresponds to the total number of eligible cases identified over the study period (June 2014 to July 2022), based on available laboratory records and the inclusion criteria.

Data collection

Data were extracted from laboratory records and the Elabs software. The data collection forms included the following information: patient identification (last name, first name, sex, and age), date of analysis, referring department, and macroscopic and microscopic results of the parasitological stool examinations. The collected data were then entered into a Microsoft Excel spreadsheet for organization and further statistical analysis.

Parasitological analysis methods

A parasitological stool examination begins with a macroscopic assessment to evaluate the sample’s appearance, consistency, color, and the presence of visible abnormalities such as blood or mucus. This step provides initial diagnostic clues before proceeding to microscopic analysis. The fresh stool is then examined under a microscope to detect motile protozoa or cysts. To enhance visualization, a staining technique is applied, commonly with 2% Lugol’s iodine solution or the Merthiolate Iodé Formol (MIF) mixture. These dyes help identify internal structures of protozoa and helminths. Following this, parasite concentration techniques are employed to improve detection sensitivity, especially when parasite load is low. In our laboratory, we primarily use the MIF method and the Bailanger method, both of which are widely recognized for their effectiveness in concentrating parasitic elements. These steps are essential for accurate diagnosis and epidemiological surveillance of parasitic infections.

Statistical analysis

The statistical analysis was performed using SPSS software. Data were expressed as counts and percentages for qualitative variables.

## Results

A total of 684 stool samples from patients diagnosed with IBD were analysed during this study. Of these, 162 tested positive, corresponding to an overall prevalence rate of 24%.

Patient demographic profile

Of the 162 patients included in the study, 159 (98%) were adults and 3 (2%) were children. In terms of gender distribution, 97 patients (59%) were female and 65 (41%) were male, giving a male-to-female ratio of 0.7.

Distribution by type of IBD

Analysis of the clinical forms of IBD revealed that 78 patients (48%) had UC, 67 patients (41%) had CD, and 17 patients (11%) had indeterminate IBD.

Distribution according to results of PES

**Figure 1:**
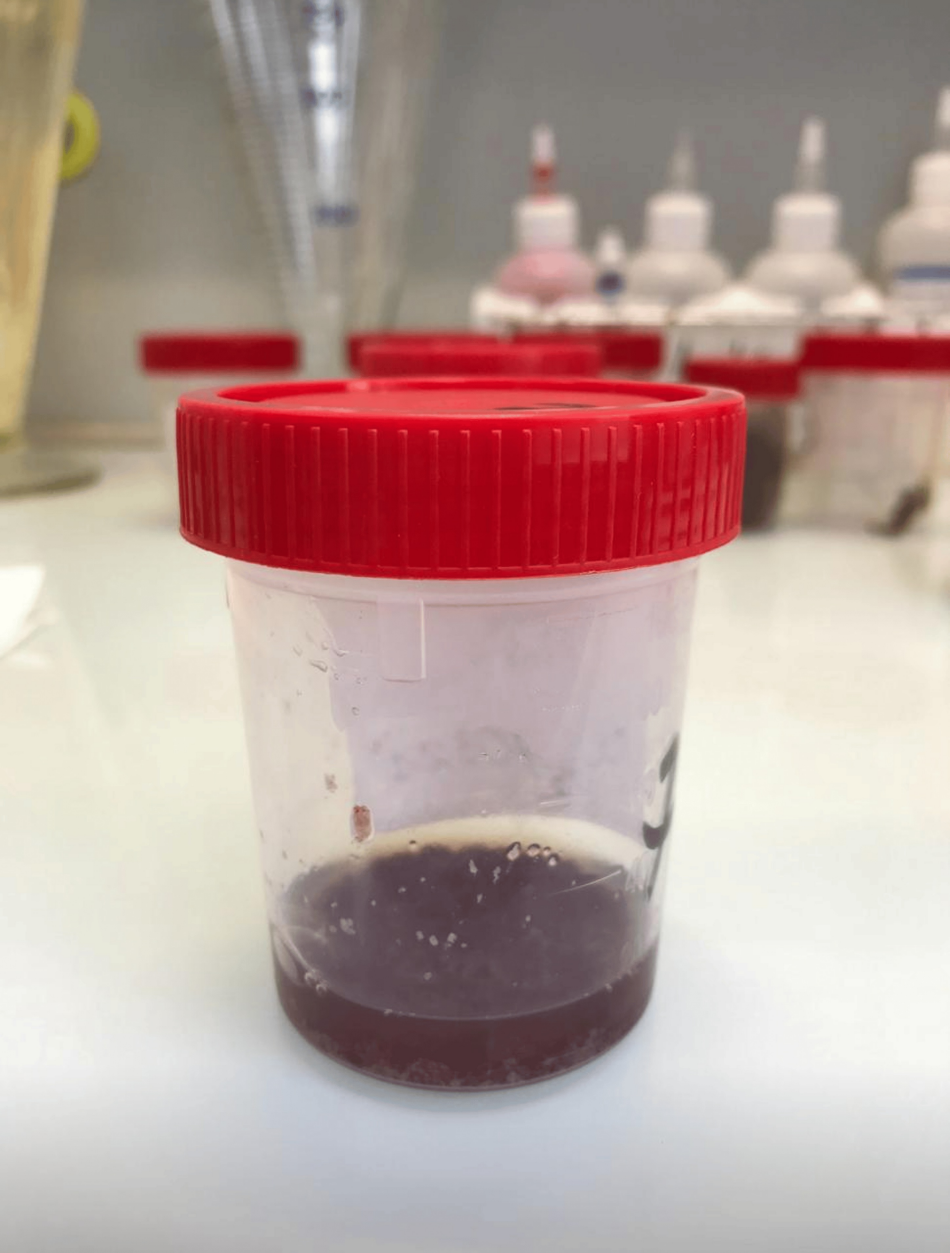
Macroscopic appearance of bloody mucus in stool.

Macroscopic examination of stools revealed that 130 patients (80%) had bloody and mucous diarrhoea. The remaining 20% had normal-appearing stools (Figure 1).

**Figure 2:**
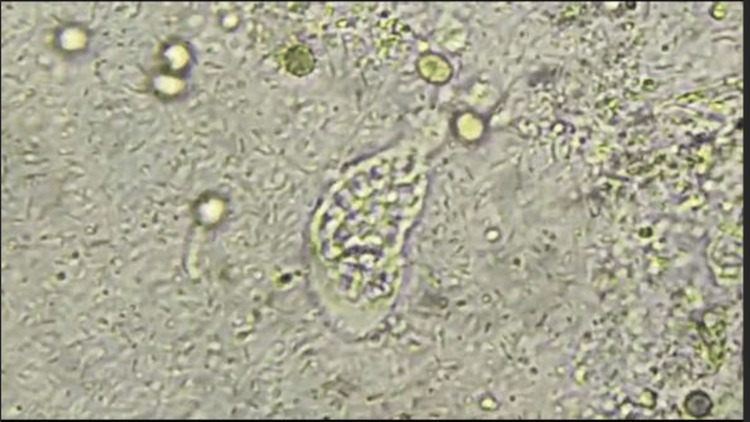
Vegetative form of Entamoeba histolytica at 40× magnification.

Microscopic examination of stool samples in our study revealed the presence of the blood-feeding vegetative form (FVHH) of Entamoeba histolytica in 98 patients (60%) (Figure 2), followed by the cystic form (KEH/KED) of *Entamoeba histolytica*/*Entamoeba dispar* in 38 patients (23%). A combination of the two forms was observed in 23 cases (14%). The *Entamoeba histolytica* minuta form was isolated in only three patients (2%).

The demographic and parasitological data of IBD patients are presented in Table 1.

**Table 1:** Demographic and parasitological characteristics of patients with IBD (n = 162). IBD: Inflammatory bowel disease.

Category	Variable	Number (n)	Percentage (%)
Parasitic Prevalence	Positive samples out of 684	162	24
Age Distribution	Adults	159	98
	Children	3	2
Sex	Male	65	41
	Female	97	59
	Male-to-female ratio	—	0.7
Type of IBD	Ulcerative colitis (UC)	78	48
	Crohn’s disease (CD)	67	41
	Indeterminate colitis	17	11
Macroscopic Stool Appearance	Bloody and mucous diarrhea	130	80
	Normal-appearing stools	32	20
Microscopic Findings	Trophozoite form of *E. histolytica*	98	60
	Cyst form of *E. histolytica/E. dispar*	38	23
	Combination of both forms	23	14
	*E. histolytica* minuta form	3	2

## Discussion

Amoebiasis is one of the most prevalent parasitic diseases worldwide, particularly in regions with poor hygiene conditions such as Africa, Southeast Asia, and Latin America [11]. In Morocco, it is a significant public health problem due to its high incidence, exacerbated by the large number of asymptomatic carriers. According to national data, the prevalence of amoebiasis ranges from 7.6% to 37%, as determined by microscopic stool examinations [12].

In recent decades, the role of amoebiasis in triggering or exacerbating IBD has frequently been discussed [13,14]. Several international studies have supported this hypothesis by showing a significant prevalence of amoebiasis in patients with IBD (Table 2) [15-17]. However, no national statistical study has yet been conducted to assess the relationship between these two diseases.

**Table 2:** Distribution of prevalence rates reported in different studies. Data adapted from references 15-17. IBD: Inflammatory Bowel Disease; UC: Ulcerative Colitis; CD: Crohn’s Disease.

Different Studies	Prevalence of Amoebiasis in Patients with IBD	Prevalence in UC Patients	Prevalence in CD Patients
Our study (8 years)	24% (162/684)	38% (78/204)	16% (67/418)
Babić E et al. (2 years, Bosnia) [15]	16% (19/119)	14.3% (12/84)	20% (7/35)
Salama RI (1 year, Egypt) [16]	18.3% (11/60)	18.4% (9/49)	18.2% (2/11)
Ustun S et al. (1 year, Turkey) [17]	8.75% (14/160)	10% (13/130)	3.3% (1/30)

In our study, the prevalence of intestinal amoebiasis was 24%, which, although significant, remains lower than the figure of 31.5% reported in another study conducted at Yuksek Ihtisas Hospital in Turkey [18]. However, this rate is higher than that observed in populations without intestinal mucosal damage, suggesting that IBD may promote the development of intestinal amoebiasis [19]. Patients with IBD may indeed be more susceptible to *Entamoeba histolytica* infection due to mucosal damage, and this infection could also exacerbate their condition. This underlines the importance of performing a PES before establishing a diagnostic plan for suspected cases of IBD.

In our series, the majority of patients were adults, predominantly female, which is consistent with data from a Tunisian study conducted at La Rabta Hospital, which also noted a female predominance [20]. This suggests the influence of hormonal and immunological factors, although the exact mechanisms responsible for this female predominance remain unclear. In contrast, a study conducted at the Mostar Clinical Hospital in Bosnia found no significant difference in the prevalence of IBD and amoebiasis between the sexes, compared to a healthy population. In this context, amoebiasis was not considered a risk factor for IBD.

When analysing patients with IBD, whether UC or CD, the association between intestinal amoebiasis and UC appears to be the most commonly documented in the literature [17,21]. Indeed, many amoebic superinfections have been mistaken for flare-ups of UC, and vice versa, due to the similarity of the clinical manifestations and endoscopic lesions of the two conditions. Similarly, in our study, the association with UC was more frequent than with CD. This is probably because CD generally affects the small intestine, whereas *Entamoeba histolytica* inhabits the colon, making an association with colonic lesions (UC) rather than small intestinal lesions (CD) more logical [16].

During the microscopic examination of stool samples in our study, the FVHH form was identified in the majority of patients with intestinal amoebiasis, accounting for 60% of cases. This finding aligns with a study conducted at the University of Minnesota in Minneapolis, USA, which found that 62% of infected individuals had this form. This highlights the prevalence of FVHH in the context of IBD and suggests a potential association with amoebic dysentery [22].

Therefore, intestinal amoebiasis must always be considered in cases of inflammatory flare-ups in patients with IBD, particularly in countries where amoebiasis is prevalent, such as ours. Distinguishing between IBD and amoebic colitis is crucial, as a late diagnosis or inadequate treatment can lead to serious complications. Indeed, the inappropriate administration of immunosuppressants in the presence of amoebic colitis can promote parasitic dissemination, carrying a high mortality risk [23]. Furthermore, co-infection with *Entamoeba histolytica* can exacerbate the clinical symptoms of IBD and alter its course. This highlights the importance of systematic screening for amoebiasis in patients experiencing flare-ups of IBD to ensure appropriate diagnostic and therapeutic management.

Although our study relies on outdated laboratory techniques, limiting its diagnostic accuracy, it remains relevant in an epidemiological context. The methods used do not permit reliable differentiation between *Entamoeba histolytica* and *Entamoeba dispar*, which are morphologically identical but non-pathogenic. Nevertheless, our approach enables us to identify patients at risk, particularly those with IBD, and prevent potential complications. These observations highlight the need for more specific, modern diagnostic tools, such as molecular biology or antigen tests, to ensure accurate species identification.

## Conclusions

IBD represents a significant public health challenge, especially in regions where amoebiasis is endemic. These two conditions are interdependent: IBD can promote the onset or reactivation of amoebiasis by disrupting the intestinal barrier or through immunosuppressive treatments. Conversely, amoebiasis can mimic or worsen IBD, complicating diagnosis. Therefore, a rigorous differential diagnosis is essential. To improve diagnostic accuracy, any patient with IBD presenting with symptoms of disease activation should undergo routine amoebic screening through thorough microscopic stool examination, particularly in endemic areas where Entamoeba histolytica is prevalent. Heightened vigilance is necessary to avoid misdiagnosis and inappropriate treatment. Although this retrospective study provides valuable insights into the relationship between amoebiasis and IBD, prospective studies using more robust diagnostic protocols are needed to strengthen conclusions and optimize patient management.
